# 40.4 LOW-DOSE RISPERIDONE TREATMENT IN ADOLESCENCE PREVENTS THE DEVELOPMENT OF NEUROINFLAMMATION IN THE MATERNAL IMMUNE ACTIVATION MODEL

**DOI:** 10.1093/schbul/sby014.167

**Published:** 2018-04-01

**Authors:** Ina Weiner, Shani Pery, Jasbeer Dhawan, Anat Biegon, Yael Piontkewitz

**Affiliations:** 1 Tel-Aviv University; 2 Stony Brook University School of Medicine

## Abstract

**Background:**

Postnatal consequences of prenatal immune activation mimic a broad spectrum of neuro-psycho-pathological features phenotypic of schizophrenia (SCZ). We previously showed that SCZ-relevant behavioral and brain structural abnormalities emerging in adult offspring of moms exposed to the viral mimic polyI:C, are prevented by treatment with the atypical APD risperidone (RIS) in adolescence, prior to the emergence of structural and behavioral abnormalities. Given the increasing centrality of neuroinflammation in SCZ and its treatment and/or prevention, here we assessed whether adolescent RIS is able to prevent neuroinflammation in the polyI:C offspring.

**Methods:**

On gestation day 15, pregnant Wistar rats were injected IV with polyI:C (4 mg/kg/ml) or saline. Pups were weaned on postnatal day (PND) 21. Preventive treatment with RIS (Janssen, Belgium; 0.045 mg/kg) was administered daily on PNDs 34–47. Offspring were sacrificed on PND48, prior to full spectrum of structural and behavioral abnormalities, or on PND90, after the emergence of structural and behavioral abnormalities. Microglial activation was assessed in ten regions (nucleus accumbens, striatum, substantia nigra, frontal, anterior cingulate and occipital cortices, dorsal hippocampus (sub-regions CA1, CA3 and dentate gyrus [DG]) and ventral hippocampus (vHPC), using quantitative [3H]PK11195 autoradiography. Another cohort of offspring underwent behavioral testing and imaging.

**Results:**

ANOVAs of [3H]PK11195 binding in offspring sacrificed on PND48 revealed no significant effects of prenatal polyI:C in any of the regions assessed. In adult male offspring, [3H]PK11195 binding was significantly increased in the CA1, CA3 and DG hippocampal subfields as well as in the frontal and occipital cortices, compared to controls. No such increases were observed in polyI:C offspring treated with RIS in adolescence (significant prenatal x preventive treatment interactions, and significant difference in [3H]PK11195 binding between polyI:C-VEH and saline-VEH but not between polyI:C-RIS and saline-VEH offspring in post-hoc analyses, in each of the regions). In females, [3H]PK11195 binding was significantly increased only in the vHPC, occipital cortex, and nucleus accumbens. Such increases were not observed in polyI:C female offspring treated with RIS in adolescence. In a second cohort of offspring, prenatal poly-I:C led to structural abnormalities in the hippocampus, striatum, prefrontal cortex and lateral ventricles, as well to deficits in selective attention, executive function, working memory and social interaction, all of which were prevented by RIS.

**Discussion:**

Increased [3H]PK11195 binding in the brains of adult poly-I:C offspring is consistent with increased uptake of [11C]PK11195 in patients with SCZ, measured in-vivo by PET. Microglial activation emerged in adulthood, with no such activation in young (PND48) offspring. Late emergence of microglial activation parallels the developmental course of behavioral and brain structural abnormalities in poly-I:C offspring (Piontkewitz et al, 2011a, 2012a; Piontkewitz et al, 2009), suggesting that these late-emerging abnormalities are linked. The latter is supported by the fact that RIS in adolescence prevented the emergence of behavioral and brain structural abnormalities as well as microgliosis in the adult offspring. These data suggest that prevention of adult microgliosis is one of the mechanisms underlying RIS capacity to prevent polyI:C-induced behavioral and neuroanatomical deficits, however, a causal relationship remains to be established.

